# Reaching out for Help: Calls to a Mental Health Helpline Prior to and during the COVID-19 Pandemic

**DOI:** 10.3390/ijerph18094505

**Published:** 2021-04-23

**Authors:** Josianne Scerri, Alexei Sammut, Sarah Cilia Vincenti, Paulann Grech, Michael Galea, Christian Scerri, Daniela Calleja Bitar, Stephania Dimech Sant

**Affiliations:** 1 Department of Mental Health, Faculty of Health Sciences, University of Malta, Tal-Qroqq, MSD 2080 Msida, Malta; alexei.sammut@um.edu.mt (A.S.); sarah.cilia-vincenti@um.edu.mt (S.C.V.); paulann.grech@um.edu.mt (P.G.); michael.galea@um.edu.mt (M.G.); 2 Faculty of Health, Social Care and Education Kingston University and St George’s University of London, Kingston-Upon-Thames KT2 7LB, UK; 3 Department of Physiology and Biochemistry, Faculty of Medicine, University of Malta, Tal-Qroqq, MSD 2080 Msida, Malta; christian.scerri@um.edu.mt; 4 Richmond Foundation 424, St. Joseph High Road, SVR 1013 St. Venera, Malta; coo@richmond.org.mt (D.C.B.); ceo@richmond.org.mt (S.D.S.)

**Keywords:** coronavirus, psychological impact, anxiety, depression, mental health

## Abstract

The COVID-19 pandemic is a major health crisis associated with adverse mental health consequences. This study examined 2908 calls made to a national mental health helpline over a 10 month period, 2 months prior to (Pre-COVID) and 8 months during the pandemic phase, that incorporated the imposition of a partial lockdown, followed by the removal and reintroduction of restrictive measures locally. Data collected included reason/s for call assistance, gender, age and number of daily diagnosed cases and deaths due to COVID-19. In the Pre-COVID phase, calls for assistance were related to information needs and depression. With the imposition of a partial lockdown, coupled with the first local deaths and spikes in number of diagnosed cases, a significant increase in number of calls targeting mental health, medication management and physical and financial issues were identified. Following the removal of local restrictions, the number of calls decreased significantly; however, with the subsequent reintroduction of restrictions, coupled with the rise in cases and deaths, assistance requested significantly targeted informational needs. Hence, whilst calls in the initial phase of the pandemic mainly targeted mental health issues, over time this shifted towards information seeking requests, even within a context where the number of deaths and cases had significantly risen.

## 1. Introduction

In December 2019, the Chinese authorities first imparted to the world the accelerated transmission of a new virus, SARS-CoV-2 [1]. This was followed on 30 January with an announcement by the World Health Organization (WHO) that this outbreak was a public health emergency of international concern, with the direct health and economic impact of the pandemic and the disruption of social and community structures increasing the likelihood of a major international mental health crisis [2]. Current data from the European Centre for Disease Prevention and Control indicates that until 31 October, 2,000,252,224 cases and 181,992 deaths were reported in the EU/EEA and the UK [3].

In an effort to flatten the pandemic curve and to avoid the collapse of overburdened health systems, governments worldwide have responded to this threat with impositions such as border shutdowns, travel restrictions, quarantine and closure of educational institutions and non-essential service outlets [4]. Anxiety, depression, increased alcohol and substance use, distress, anger, insomnia and increased risk of suicide have been reported, whilst risk factors for mental disorders such as loneliness and domestic and physical violence have also been highlighted. Individuals with existing mental health challenges have been identified as being particularly affected due to a lack of continued psychiatric care services [5,6]. Furthermore, the pandemic has put a greater strain on the mental health of various groups including homeless persons and migrant workers who have been rendered more vulnerable due to socioeconomic repercussions. Many healthcare workers have experienced significant fears of contagion and of spreading the virus to their families, friends or colleagues. Additionally, as highlighted in prior research on such severe epidemics, it is expected that the mental health impact of the current pandemic is not short term but rather is likely to continue for a long period even after the pandemic ends [7].

Within this scenario a dire need for safe communication channels targeting mental health challenges has arisen. The restrictions of face-to-face contacts have identified mental health helplines as one such channel. Such helplines enable the person to log in daily and make timely contact with support services. This can mitigate isolation, as well as address themes of fear, uncertainty and stigmatisation. Despite the significant impact of such helplines, there is a dearth of research on the impact of the COVID-19 scenario on calls made to mental health helplines, with five studies being extracted from the general literature. Three of these studies [8,9,10] report the activity of mental health helplines specifically set up as a response to the pandemic in Greece, India and Nepal, respectively. Peppou et al. [8] analysed data from a random sample of 576 calls out of a total of 1728 calls made to a nationwide helpline over a 3-week period following the outset of restrictive measures, whilst Ravindran et al.’s [9] study examined data from 20,475 calls during the four weeks when the country was in the state of lockdown. Both studies highlighted the initial preponderance of anxiety symptoms and demonstrated that the majority of calls comprised concerns specific to COVID-19, such as fear of the illness and issues relating to quarantine. Whilst Peppou et al.’s [8] study revealed a greater percentage of callers identified as having clinically important depression (i.e., 37%) versus that for anxiety (i.e., 20.3%). Correspondingly, Ravindran et al. [9] highlighted that most of the initial calls were related to anxiety, but over time people were calling because they were grappling with depression and substance abuse. Conversely, in another study by Shakya [10], 102 calls made to a helpline run by a single centre over a one-week period were examined. The major concerns of callers revolved around closure of services and unavailability of medications and only 3.33% of calls related to fear of the coronavirus. Two other studies [11,12] examined symptom profiles and concerns of callers to nationwide established helplines in Switzerland and Australia, respectively, both prior to and during the pandemic. With regard to symptom profiles, Titov et al. [12] observed a small but significant increase in anxiety symptoms but no differences in levels of psychological distress, depression or suicidal thoughts, whilst Brulhart and Lalive [11] identified small increases in loneliness and struggles attributed to life adjustments during lockdown. In their study, Brulhart and Lalive [11] also identified the need for research exploring trends in helpline calls made as lockdown restrictions were lifted. 

Hence, this study contributes to extant literature by exploring local trends in helpline calls within the following contextual phases:

(i) ‘Pre-COVID’ (i.e., January–February 2020) prior to the local pandemic;
(ii) Partial lockdown (i.e., March–April 2020) that included a ban on all sea and air travel (except for cargo), a mandatory 14-day quarantine on travellers returning from various countries and the closure of schools, day centres for the elderly and non-essential retail and services;
(iii) Partial easing of restrictions (i.e., May–July 2020) with the opening of non-essential retail and services, the re-opening of the airport and the organisation of mass events in the entertainment industry;
(iv) Reintroduction of restrictions (i.e., August–October 2020) that included the banning of mass gatherings and the enforcement of compulsory wearing of masks in all public spaces, following a spike in confirmed cases with COVID-19.

To examine these trends in helpline calls, the following research questions were addressed in the present study: (i) How do the number of monthly calls made to a mental health helpline vary over a 10 month period, incorporating a Pre-COVID and COVID phase?; (ii) Does the number of monthly diagnosed COVID-19 cases and deaths relate to the number of monthly calls made to the helpline?; (iii) What are the main presenting reason/s for call assistance over time?; (iv) How do the number of monthly calls relating to personal (versus ‘others’) issues and targeting COVID-19 queries vary over the 10 month period? 

The following research hypotheses were then generated that address the research questions set: (i) the number of monthly call requests for assistance will vary over time, increasing during the COVID phase; (ii) there will be a greater number of calls for assistance during months when there is a rise in the number of COVID-19 cases and deaths; (iii) the main presenting reasons for call assistance during the COVID-19 phase will relate to mental health illnesses and social connectiveness issues such as loneliness and (iv) a greater number of calls will relate to personal issues, whilst assistance regarding COVID-19 queries will be significantly higher in the initial COVID phase (i.e., March, April, May) when the population is encountering a novel challenge. 

## 2. Materials and Methods

### 2.1. Participants

Based on data published by the National Statistics Office, the current population of Malta is 514,564 [13], consisting of 265,762 males and 248,802 females. The population in this study comprised all people who requested mental health assistance via phone to a non-governmental organisation (namely Richmond Foundation) who managed the national mental health helpline. A total of 2908 requests for help were made between January to October 2020. Sixty-two percent of requests for assistance were made by females. The modal age category was that of 66 years and above. However, this finding should be treated with caution as 42.4% of the participants did not provide details regarding their age.

### 2.2. Data Collection 

Helpline assistance was provided by a range of mental health professionals, namely psychology graduates; psychotherapists, social workers and mental health occupational therapists who would have all undergone specialised training delivered by a clinical psychologist. The role of such operators was to document details pertaining to demographic characteristics of participants (i.e., gender, age), date of call, reason/s for requesting mental health assistance, if the call was COVID related and whether for gender or personal reasons or regarding the welfare of others. Furthermore, this information was augmented with a brief textual description of each presenting case documented by the operator both during and following the call. This textual information was further reviewed by the first (J.S.) and second author (A.S.) and compared to the data inputted into the system. Thus, for example, a call was determined to be COVID-19 related if it was either specifically cited by the person requesting assistance or whether the textual account presented indicated that the caller was requesting mental health assistance due to COVID-related aspects, such as the partial lockdown and/or quarantine amongst others. 

Furthermore, to ensure that all requests for assistance were adequately considered and targeted, the number of operators providing the service was influenced by the number of calls being received through the helpline. 

Data relating to number of cases and deaths on a daily basis were also obtained from information published by the European Centre for Disease Prevention and Control [3]. 

### 2.3. Data Analysis

Statistical analysis was conducted using SPSS version 26. Percentages of responses were calculated according to the number of respondents per response with respect to the number of total responses to a question. Statistical significance relating to categorical variables was determined using the one variable Pearson’s chi-square test. For the null hypothesis to be true, it was expected that frequencies would be equally distributed by month. A 0.05 level of significance was applied to determine statistical significance.

### 2.4. Ethical Issues

Ethical approval was obtained from the relevant research ethics committee at the University of Malta (Proposal number: V_11022020 5625). Anonymised data were provided by the non-governmental organisation running the national mental health helpline to the researchers and hence no persons were identifiable.

## 3. Results

The demographic and COVID-related characteristics for the study population are presented in Table 1. A significant association between the number of calls by month (χ^2^ (9, N = 2908) = 2298.14, *p* ≤ 0.001) was obtained with a greater number of calls recorded over the months of April (*n* = 973; 33.4%), May (*n* = 494; 17.0%), August (*n* = 311; 10.7%) and October (*n* = 328, 11.3%). From a total of 2908 calls, 62% (*n* = 1803) were females. Approximately half of the calls made over two months, namely April (56%) and May (43%), were COVID-19 related. During the Pre-COVID versus COVID phase, 57% versus 80.9% of calls, respectively, were for personal reasons. The first local deaths due to COVID-19 were reported during the month of April (*n* = 4) with the modal category for number of monthly deaths being that for October with 27 deaths. A significant association was detected for number of COVID-19 cases by month (χ^2^ (7, N = 2908) = 9502.1, *p* ≤ 0.0001), with a greater number of cases diagnosed for the months of August (*n* = 1144), September (*n* = 1178) and October (*n* = 2984).

**Figure 1:**
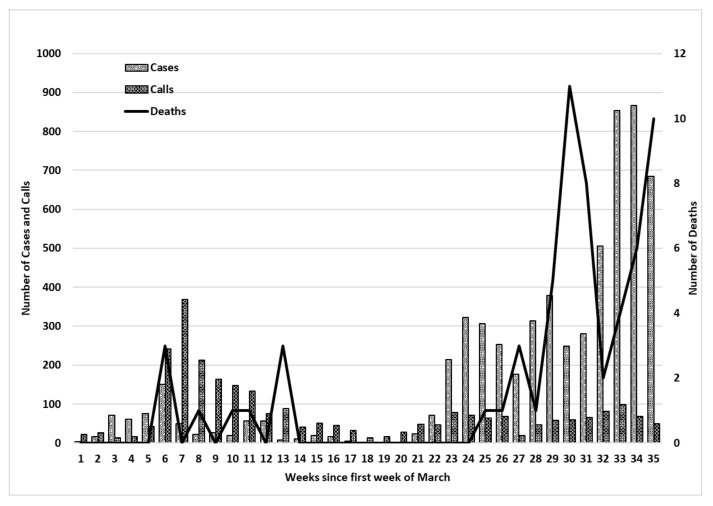
Number of calls to a helpline, cases and deaths over a 35 week period. N.B.: Data collected from daily publication of data by the European Centre for Disease Prevention and Control.

Figure 1 presents data relating to the weekly number of: (i) helpline calls, (ii) cases diagnosed with COVID and (iii) deaths between the months of March and October 2020. Week 1 corresponds to the first week of March and week 35 corresponds to the week ending 31 October. In March, 86 calls were made to the helpline amounting to 2.9% of the total number of calls made. During this month, no deaths from COVID-19 were registered and the total number of cases diagnosed was of 169 cases, with a maximum of 17 cases on two -consecutive days, 22 and 23 March 2020. The month of April accounted for 33.4% (*n* = 973) of the total amount of calls made to the helpline of which 62.8% (*n* = 611) were made during weeks 6 and 7 (i.e., between 6 April and 19 April). This period corresponded to the: (i) first local deaths registered on the 9, 10 and 12 April and (ii) an increase in the number of cases during week 6, with two spikes in individuals diagnosed with COVID-19 on 7 (*n* = 52) and 9 April 2020 (*n* = 38).

Between the months of May and July (i.e., weeks 10 to 21), there was a decrease in the number of helpline calls (May: 494; June: 182; July: 127), cases with COVID-19 and deaths. This corresponded to a period when restrictions were gradually eased and eventually removed. Between August and October, an increase in the number of persons diagnosed with COVID-19 (i.e., August: *n*= 1144; September: *n* = 1178; October: *n*= 2984) and in the number of deaths was noted. Yet although the number of calls for assistance between August and October (i.e., August: *n*= 311; September: *n*= 204; October *n*: 328) did increase in comparison to the months of June and July, the levels attained did not reach those recorded during the initial stage of the COVID-19 pandemic (i.e., April: *n* = 973; May: *n* = 494).

Table 2 presents the 10 most highly ranked causes for assistance from the mental health helpline. The highest ranked causes for assistance in the Pre-COVID phase (i.e., January–February) were information needs relating to mental health and available services (*n* = 60), depression (*n* = 59) and psychological distress (*n* = 51). Between the months of March and June, the highest frequency of calls related to mental health issues, namely anxiety, psychological distress, depression and loneliness. This period corresponded to the imposition of a partial lockdown followed by some easing of restrictions in May. For the months of July–August, the main reasons for call assistance related to informational needs (*n* = 128), loneliness (*n* = 92), psychological distress (*n* = 92) and depression (*n* = 92), whilst for September–October, assistance was requested mainly due to informational needs (*n* = 251), psychological distress (*n* = 114), anxiety (*n* = 98), depression (*n* = 89) and loneliness (*n* = 89).

An analysis of trends in calls made over time in months (Table 2) indicated a significant number of calls between March and June relating to anxiety (χ^2^ (4, N = 2908) = 473.7, *p* < 0.0001), depression (χ^2^ (4, N = 2908) = 117.5, *p <* 0.0001), financial reasons (χ^2^(4, N = 2908) = 85.7, *p* < 0.0001), medication management (χ^2^ (4, N = 2908) = 74.2, *p* < 0.0001), psychological (χ^2^ (4, N = 2908) = 363.9, *p* < 0.0001), physical health (χ^2^ (4, N = 2908) = 86.5, *p* < 0.0001) and loneliness (χ^2^ (4, N = 2908) = 196.5, *p* < 0.0001). Calls relating to family issues (χ^2^ (4, N = 2908) = 40.7, *p* < 0.0001) and obsessive compulsive disorder (χ^2^ (4, N = 2908) = 26.4, *p* < 0.0001) were significantly higher between March and June and September and October, whilst calls related to suicidal issues were higher between March and July (χ^2^ (4, N = 2908) = 12.6, *p* < 0.001). Requests for assistance relating to informational needs were significantly higher between September and October (χ^2^ (4, N = 2908) = 129.3, *p* < 0.0001).

## 4. Discussion

### 4.1. Significance of the Study

Extant literature, such as cross-sectional surveys exploring population mental health during the COVID-19 outbreak, are characterised by the use of convenience samples, lack of standardisation of measures, lack of comparable pre-COVID-19 baseline data and often a focus on specific subgroups, such as students or healthcare workers. The present study contributes to this literature by analysing national helpline data over a 10-month period that includes comparative data from the ‘Pre-COVID’ and ‘COVID phase’, respectively. The decision to examine helpline data was influenced by its advantage as a research method as it provides a more reliable gauge of the prevalence and monitoring of distress and is in real time, as calls are logged in daily [11]. Additionally, the role of these helplines is expected to increase, and consequently, research relating to the utilisation of helplines is highly relevant.

The present study further contributes to extant literature by embedding and interpreting the data collated within a contextual framework that includes information relating to the number of deaths and cases diagnosed with COVID-19 and within different phases of the pandemic; namely, a partial lockdown, the gradual easing of restrictions and during a second extensive wave when restrictions were reintroduced. Such information is of importance considering that this outbreak is ongoing, and the mental health impact of the pandemic is also likely to prevail long after the pandemic ends [5]. The present study highlights that although pre-existing counsellors in the community continued to provide their services either face-to-face (especially for persons with severe mental illnesses) and/or online during the COVID phase, yet as hypothesised, requests for helpline mental health assistance varied over time, with an increase in calls detected during the months of April and May (i.e., corresponding to spikes in COVID cases and the first deaths) and between August and October (i.e., corresponding to increases in the number of cases and deaths at the beginning of the second wave). Further ongoing research is required, however, on the mental health needs and concerns of people, as countries grapple with second and consecutive waves of COVID-19. Such information can provide a reference that can inform the development of responses in the eventualities of other waves of COVID-19 and may crucially inform any relevant interventions that can be formulated [14]. For instance, the present study has demonstrated that calls relating to family-related issues rose significantly during the initial months of the first wave and then again during the second wave, whilst requests relating to informational needs rose significantly during the second wave (i.e., September and October 2020). Hence, there is a need for governments to ensure that the mental health budget allocation adequately covers the increased burden being experienced by families such as care provision to the vulnerable that includes the elderly, persons with a mental illness and parents caring for a child with behavioural, psychological and/or physical challenges, amongst others. Furthermore, decision makers and helpline agencies should focus on strengthening community care services, such as through the training and upskilling of staff, that can target the increased mental health concerns and informational requirements of people. In addition, the application of technology-enabled services such as e health, m health and telemedicine provides the availability of online-based learning and self-help services, the opportunity for person-to person communication in real time whilst maintaining social distancing and the linking of informational requests to services available. Further research, however, is required to evaluate such services whilst determining the most effective means to target the provision of information to persons, especially the most vulnerable populations. 

### 4.2. Number and Types of Requests Made to the Mental Health Helpline

Previous research outlines that COVID-19-related calls made up an extensive proportion of the total number of calls during the outbreak [8,9,11,12]. An exception, however, is the study by Shakya [10] who reports that the number of calls expressing concern over service closures and unavailability of medicines grossly outnumbered COVID-19-related calls. The author suggests this is explained by Nepal’s collapse of health services due to the poor development index status. In the present study, however, an increase in the number of COVID-19-related calls was identified in the month of April 2020, during which a partial lockdown was still imposed, coupled with the recording of the first deaths and spikes in number of persons diagnosed with COVID-19. However, the number of local calls for assistance also rose significantly during the months of August and October. This could be attributed to the local increase in persons diagnosed with COVID-19 following a series of mass events held in August, whilst in October, both the number of cases (*n* = 2984) and the number of local deaths (*n* = 27) rose. Additionally, in alignment with extant literature [10,15,16], people were more likely to call discussing their concerns rather than those of others. Possibly when people are too distressed themselves, their capacity to look after others may be somewhat affected. 

In relation to gender, findings from the present study indicate that during the COVID phase overall, a slightly greater percentage of females made use of the helpline for mental health assistance. Various studies [12,17,18] highlight that women were identified as more vulnerable to psychological distress during the pandemic and may be more at ease discussing their vulnerabilities than males. In fact, according to Pierce et al. [17], males might be less willing to engage in help-seeking behaviours than women and may resort to other means of targeting mental distress, such as through the abuse of alcohol. The influence of another demographic variable—that of age—on requests for helpline assistance could not be reliably determined as 42% of callers chose not to provide such details.

Call volume findings from this study resonate with published literature. Irrespective of the country of origin, methodology employed (cross-sectional surveys or analysis of helpline data) and measures, authors concur that psychological distress levels and loneliness increased considerably at the outset of the outbreak when heavy restrictions were imposed. Whilst such restrictions help to curtail the spread of infections, changes in everyday routine and a reduced access to family, friends and other social support systems may place persons at risk of psychological harm [19,20]. However, the present study contributes to extant literature by demonstrating that during the partial lockdown period and with the gradual easing of some restrictions (between April and June 2020), calls for assistance pertaining to mental health aspects (namely anxiety, depression, psychological distress, loneliness), financial problems (e.g., due to reduced working hours), medication management issues and physical health (e.g., sleep disturbances, chest pain, nausea) were significantly higher. This contrasts with calls made in the Pre-COVID phase that were significantly less in number and where calls for assistance pertained mainly to information needs and depression. The month of July was associated with a removal of social restrictions and the previous sharp increase in calls for assistance decreased and was noted to stabilise. A stabilisation in relation to number of calls over time also concurs with research conducted by Qiu et al. [18]. However, whilst we hypothesised that calls made throughout the COVID-19 period would relate to mental health challenges, we actually identified a decrease in calls requesting mental health assistance over the months of August–October (even though the number of deaths and cases rose). This may be attributed to the maintenance of social connectedness between individuals (albeit with some restrictions) and/or the use of denial as a means of coping with post-traumatic morbidity [21] as it reduces a sense of powerlessness during overwhelming stress. One can postulate that people in Malta were so overwhelmed psychologically during the first wave and subsequent partial lockdown that denial of the gravity of the situation is accounting for the stabilised levels of anxiety and distress in spite of a second extensive wave. Wang et al. [16] also posit that additional information on vaccines and an increased frequency of wearing masks also relate to improved mental health in persons.

Conversely, although calls relating to mental health issues remained stabilised, requests relating to OCD, suicidal thoughts, family-related issues and information requests (although present between March and June) were also significantly present over the months of September and October. Informational needs spiked in October, with many of the responses relating to queries regarding services and support available and aspects relating to COVID-19. The provision of information about the availability of services, such as those relating to counselling, helplines, respite services, rape crisis centres and telehealth should be made known to the general public though various sources, amongst which are mainstream channels, social media and health facilities [20].

### 4.3. Strengths and Limitations

The time frame during which call data were collected encompassed a pre COVID-19 comparison period, the outset of the outbreak, a partial lockdown, a remission phase with easing of restrictions and a second extensive wave. This allowed the juxtapositioning of call volume and reasons for call assistance along the chronicle of the COVID-19 outbreak in Malta. Data from all the 2908 calls made to the helpline during this time frame were collected in real time, thus circumventing methodological issues related to representativeness of random samples and retrospective analysis of records or reports.

Since 42% of callers chose not to give details about their age, and data such as psychiatric premorbidity and socioeconomic determinants were not collected, inferences could not be made about call assistance for some vulnerable groups identified by previous research. As with all other research analysing helpline data or cross-sectional surveys employing self-report questionnaires, one must keep in mind that self-reported levels of psychological distress, anxiety and depression may not always be in alignment with assessments made by clinicians.

### 4.4. Implications and Recommendations

It is clear from this study that trajectories for call assistance are varying over time in response to the COVID-19 pandemic. For instance, this study has demonstrated that call requests relating to family issues were significantly higher at the beginning of the first and second waves of COVID-19, whilst informational needs increased significantly during the beginning of the second wave (i.e., September–October 2020). Consonant with previous studies, females have also self-reported psychological distress, anxiety and depression to a slightly higher extent than males. It is imperative to explore whether men have experienced less distress or whether it is going unnoticed and to make sure services offered are socially acceptable to men. 

More than ever research now needs to focus on finding innovative ways of tapping on technology to develop interventions and facilitate service delivery. In fact, the importance of mental health helplines and telemedicine has been ramped up during this pandemic and it is anticipated to heighten in tandem with an increased reliance on the digital world. This reality poses challenges, however, such as a reduced ability to gauge body language and to effectively respond to acute psychiatric emergencies over the phone. Moreover, challenges pertaining to accessibility to all segments of the population, acceptability of these innovations by the general public and availability of resources to meet this demand need to be researched and subsequently addressed. Furthermore, the gauging of client satisfaction with mental health helplines and the use of interventions such as emotional freedom techniques constitute aspects that future research may address.

Mental health professionals manning such helplines can also provide support by exploring the needs and concerns of persons requiring assistance, listening empathetically and without judgment, providing first-line psychological support, motivating the public to adhere to health promotion recommendations, teaching healthy coping mechanisms, attending to their own pandemic-related distress and providing mental health care to other frontliners.

## 5. Conclusions

The present study has demonstrated that even though the number of deaths and cases had significantly increased in the latter months during the second COVID-19 wave, persons were significantly focusing on seeking information during adversity as a means of coping and possibly as a means of avoiding the mental health struggles that may have burdened them in the early months of COVID-19. Coordination between relevant authorities is necessary to ascertain that information is timely, accurate and evidence-based. Moreover, such information should also target needs and concerns that are extracted from diverse sources including helplines.

Moreover, it is paramount to brainstorm initiatives that could be introduced in the eventuality of another lockdown, either during this ongoing pandemic or any future ones. These initiatives include helping people stay connected, developing online social networks, provision of more helplines and recruiting of volunteers to support vulnerable persons.

## Figures and Tables

**Table 1:** Trends in number of calls by month.

Call Characteristics Months	Jan	Feb	Mar	Apr	May	Jun	Jul	Aug	Sep	Oct
Calls (*n*) % by month	103 (3.5)	100 (3.4)	86 (2.9)	973 (33.4)	494 (17.0)	182 (6.3)	127 (4.4)	311 (10.7)	204 (7.0)	328 (11.3)
Gender (% Female)	74 (71.8)	65 (65.0)	51 (59.3)	600 (61.7)	323 (65.4)	103 (56.6)	82 (64.6)	171 (55.0)	118 (57.8)	216 (65.9)
COVID Related (%)	0	0	31	56	43	21	20	12	20	25
Personal Reason%	67	47	74	84	79	81	74	77	84	81
Number of COVID-related deaths/month	0	0	0	4	5	0	0	3	22	27
Number of COVID cases/month	0	0	169	296	153	52	67	1149	1164	2983

Jan = January; Feb = February; Mar = March; Apr = April; Jun = June; Jul = July; Aug = August; Sep = September; Oct = October.

**Table 2:** Trends in calls for assistance made during the Pre-COVID and COVID phase.

Requests for Assistance\Month		Jan–Feb	Mar–Apr	May–Jun	Jul–Aug	Sep–Oct
Anxiety (*n* = 730)	O	39	369	153	71	98
	E	146	146	146	146	146
Depression (*n* = 569)	O	59	210	119	92	89
	E	113.8	113.8	113.8	113.8	113.8
Psychological Distress (*n* = 831)	O	51	357	217	92	114
	E	166.2	166.2	166.2	166.2	166.2
Loneliness (*n* = 569)	O	11	210	119	92	89
	E	104.2	104.2	104.2	104.2	104.2
Family Issues (*n* = 351)	O	27	92	87	62	83
	E	70.2	70.2	70.2	70.2	70.2
OCD (*n* = 102)	O	4	35	25	15	23
	E	20.4	20.4	20.4	20.4	20.4
Physical Health (*n* = 152)	O	9	70	42	12	19
	E	30.4	30.4	30.4	30.4	30.4
Medication Management (*n* = 136)	O	8	63	35	17	13
	E	27.2	27.2	27.2	27.2	27.2
Financial (*n* = 86)	O	2	47	26	4	7
	E	17.2	17.2	17.2	17.2	17.2
Suicide (*n* = 112)	O	10	26	31	27	18
	E	22.4	22.4	22.4	22.4	22.4
Information Needs (*n* = 726)	O	60	147	140	128	251
	E	145.2	145.2	145.2	145.2	145.2

N.B.: ‘O’ = observed frequency; ‘E’ = expected frequency; Pre-COVID phase: January–February 2020; COVID phase: March–October 2020.

## Data Availability

The data presented in this study are available on request from the corresponding author.
